# The Vulnerability of Qilian Juniper to Extreme Drought Events

**DOI:** 10.3389/fpls.2019.01191

**Published:** 2019-09-27

**Authors:** Xiaofeng Wang, Bao Yang, Fredrik Charpentier Ljungqvist

**Affiliations:** ^1^Key Laboratory of Desert and Desertification, Northwest Institute of Eco-Environment and Resources, Chinese Academy of Sciences, Lanzhou, China; ^2^University of Chinese Academy of Sciences, Beijing, China; ^3^Department of History, Stockholm University, Stockholm, Sweden; ^4^Bolin Centre for Climate Research, Stockholm University, Stockholm, Sweden

**Keywords:** Qilian juniper, vulnerability, drought, age effect, elevation, global warming

## Abstract

Identifying which trees are more vulnerable to extreme climatic events is a challenging problem in our understanding of forest and even ecosystem dynamics under climate change scenarios. As one of the most widely distributed tree species across the arid and semi-arid northeastern Tibetan Plateau, Qilian juniper (*Juniperus przewalskii* Kom.), is the main component of the local forest ecosystem, providing critical insurance for the ecological security of the surrounding areas. However, this species’s ability to cope with climate extremes (especially drought) has not been adequately assessed. Here, we apply a dendroecological approach that considers indices of resistance and resilience to quantify the vulnerability of Qilian junipers to the extreme drought events of 1957, 1966, 1979, and 1995. A total of 532 Qilian juniper trees from different age stages (100–1,100 years) and altitudes [3,500–4,000 m above sea level (a.s.l.)] were studied to assess their response characteristics during these four drought extremes. We conclude that drought extremes have a significant negative impact on the growth of Qilian juniper. The oldest Qilian junipers at the lower altitudes constituted the most vulnerable populations across the northeastern Tibetan Plateau and were characterized by the lowest resistance values, the narrowest annual rings, and the highest proportion of missing rings during the four drought years. Tree resilience after droughts was strongly related to the intensity of the drought event and did not change with tree age or elevation. A threshold of tree tolerance to drought may exist, with the more vulnerable tree individuals (e.g., the oldest Qilian junipers from lower altitudes) being exposed to the highest mortality risk when drought intensity exceeds the threshold value. Such a threshold needs further consideration, through the study of trees that have died (or are about to die) due to extreme droughts.

## Introduction

Droughts can reduce tree growth and forest productivity through changes in photosynthesis rate (Grassi and Magnani, 2005; Hinckley et al., 1979), carbon assimilation (Chaves, 1991; Lawlor and Cornic, 2002), phenology (Misson et al., 2011), tree morphology (Abrams et al., 1992; Aspelmeier and Leuschner, 2006), and others, with adverse impacts on ecosystem stability. Severe droughts may push tree growth decline beyond its biological thresholds (Folke et al., 2004), triggering widespread tree dieback (Camarero et al., 2015; Hoffmann et al., 2011) and even tree mortality (Allen et al., 2010; Anderegg et al., 2013). However, tree growth performance during extreme drought episodes is complicated and varies with species, age, size, population features, and the geographical distribution of the trees (Gazol and Camarero, 2016; Lebourgeois et al., 2013; Merlin et al., 2015; Vitali et al., 2017), which limits our understanding of the mechanism of tree response to drought. Research is increasingly focusing on areas threatened by severe droughts and heat waves to investigate drought damage based on the diverse physiological and distribution characteristics of trees (Brouwers et al., 2013; Rennenberg et al., 2006; Tyree et al., 1994) with the aim of assessing the reaction of these trees to drought events. Nonetheless, our knowledge of tree response to drought extremes is still limited, especially in some long-standing vulnerable and sensitive environments such as the northeastern Tibetan Plateau (NETP).

Qilian juniper (*Juniperus przewalskii* Kom.) is the main component of natural forests on the NETP and is widely distributed up to elevations of 3,500–4,100 m above sea level (a.s.l.) on the sunny and partly sunny slopes (Shao et al., 2005) of the Gobi Desert margins. Although their distribution is relatively scattered, it is one of the most critical components of the local fragile terrestrial ecosystem due to its wide distribution and the high number of trees, which play a crucial role in maintaining ecological stability and preventing desert expansion or erosion. However, these trees are threatened by insufficient water supply. Many dendrochronological studies conducted in this region have consistently found that radial growth of Qilian juniper is primarily limited by water shortage (Gou et al., 2013; Gou et al., 2015; Qin et al., 2015), and growth decline and mortality related to droughts have also occurred recently (Fang et al., 2015; Yu et al., 2015; Liang et al., 2016a), despite slight increases in annual precipitation during the past few decades (Li et al., 2008; Shi et al., 2007). When the wetting trend terminates, or if a transition to drier conditions occurs due to rising global temperatures (similar to other parts of inner Asia), the forests in this region will immediately be exposed to more severe water deficits than at present, exposing those trees with low drought resistance and resilience to a greater risk. Although many studies have considered the relationships between radial growth of Qilian juniper and the climate conditions on the NETP (Liu et al., 2009; Shao et al., 2005; Shao et al., 2009; Shao et al., 2010; Yang et al., 2013;Yang et al., 2014;Yang et al., 2017a;Yang et al., 2017b;Yang et al., 2019; Zhang et al., 2003), most focus on the reconstruction of paleoclimatic conditions. Fang and Zhang (2018) explored the resilience of Qilian juniper after four extreme drought events on the NETP and concluded that tree resilience to drought has increased over the past few decades. Unfortunately, their study did not involve the differentiated response of tree individuals to drought extremes, and the ability of trees to cope with drought remains insufficiently known. We now need to compare how the responses to hydraulic deficits of trees under different site conditions (e.g., altitude) and within different age bands vary, and identify which trees are more resistant and resilient to water shortage, making them more likely than other trees to survive after drought extremes.

To answer these questions, we studied 532 Qilian juniper trees from diverse age stages (100–1,100 years) and altitudes (3,500–4,000 m a.s.l.) to explore the variance in tree vulnerability to extreme droughts and to assess the main factors influencing the ability of trees to resist drought events. More specifically, the resilience indices presented by Lloret et al. (2011), which measure tree resilience, resistance, and recovery using ring width data from individual trees, were adopted to quantify the vulnerability characteristics of trees suffering from drought extremes in 1957, 1966, 1979, and 1995. The generalized linear model and superposed epoch analysis (SEA; Chree, 1913; Chree, 1914; Lough and Fritts, 1987) are used to verify the reliability of the initial results obtained in our study.

## Materials and Methods

### Tree-Ring Data and Dendroecological Analysis

**Figure 1:**
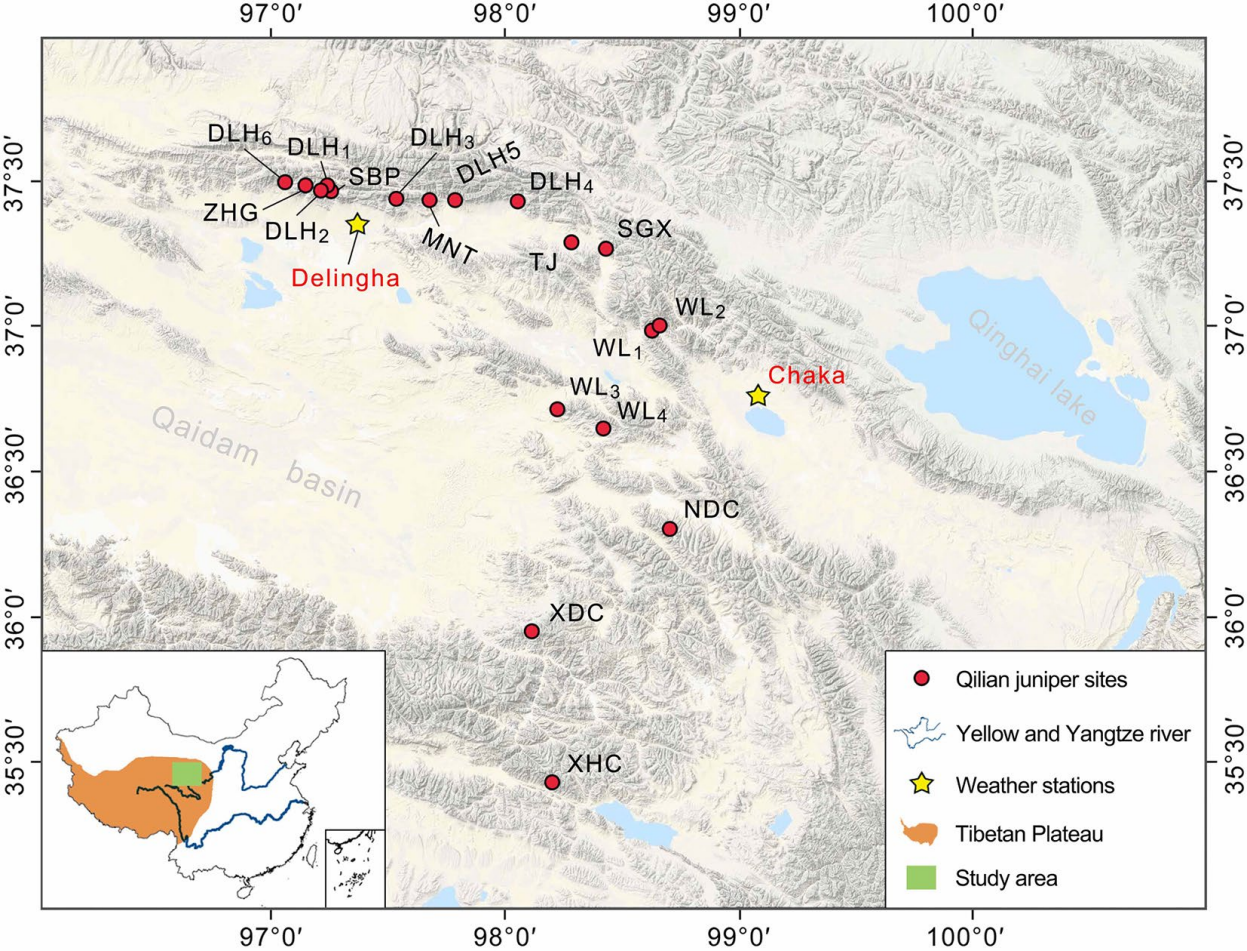
The location of the study area and sampling sites. The Qilian juniper site names and other geographic information refer to Shao et al. (2009) and Yang et al. (2014).

**Figure 2:**
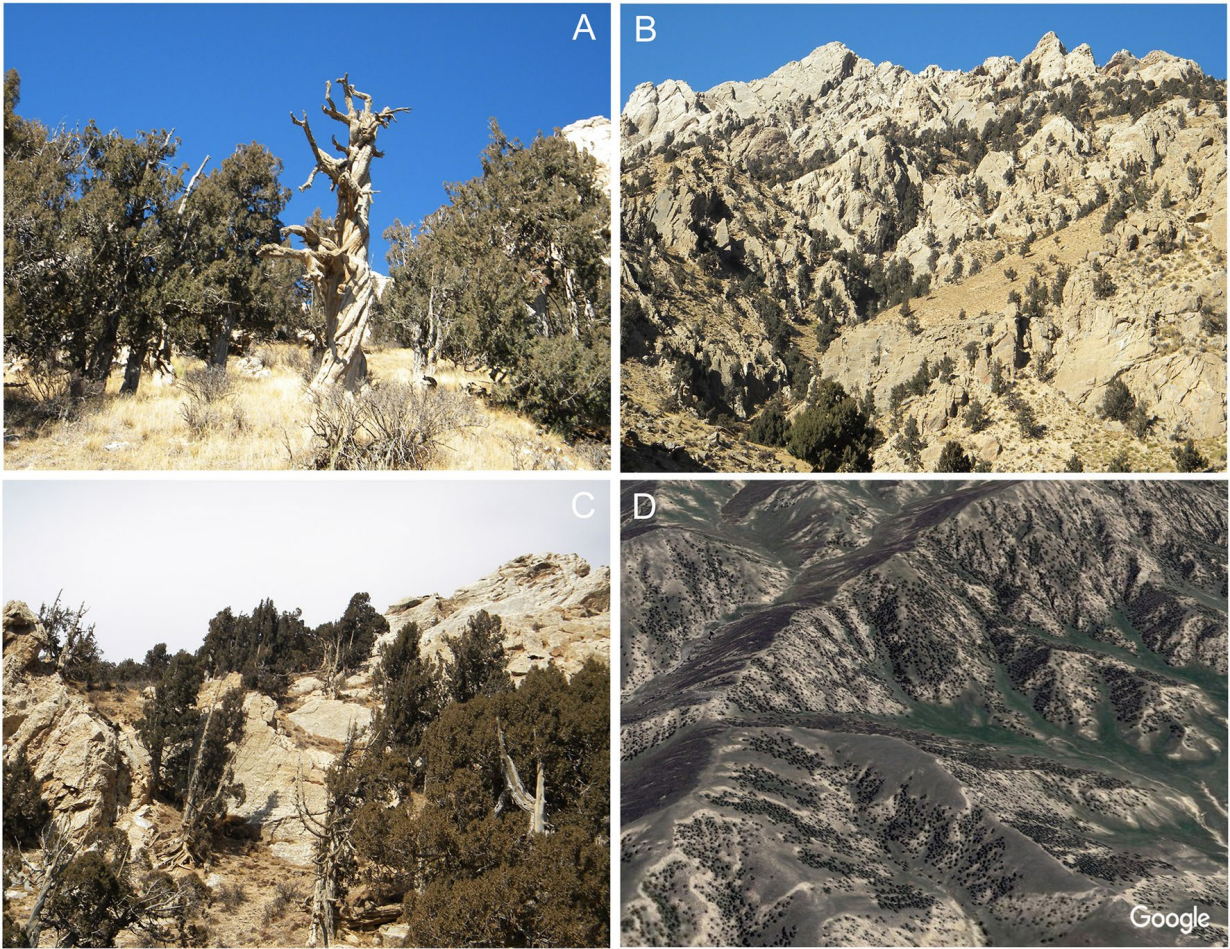
An overview of the distribution characteristics of Qilian junipers in the study area. The typical Qilian juniper individuals on the NETP **(A)** and their barren growth environment and dotted distribution **(B**, **C**, **D)**.

In order to investigate the possible disparate responses among different Qilian juniper individuals under drought stress, we re-examined published data from the NETP and classified tree-ring width series according to the following tree age criteria: age class 1 (AC1), 100–300 years; age class 2 (AC2), 301–500 years; age class 3 (AC3), 501–700 years; age class 4 (AC4), 701–900 years; and age class 5 (AC5), 901–1,100 years. We divided each age class into high altitude (3,800–4,000 m a.s.l.) and low altitude (3,500–3,700 m a.s.l.). Data from a total of 532 trees of Qilian juniper were assimilated from reported studies (Shao et al., 2009; Yang et al., 2014), distributed across 18 sites in Wulan, Delingha, Tianjun, and Dulan (**Figure 1**; **Table 1**). To maintain consistency among all tree samples, we used only one increment core per tree; thus, the mean ring width sequence was used for trees having two cores. Due to the high altitude, the cold and dry climate, and the barren soil, other tree species generally do not thrive, such that almost all of the trees growing on the NETP are Qilian junipers. These scattered trees, together with alpine meadows and shrubs distributed between the bare rocks or in the lower valleys, constitute the local plant cover (**Figure 2**). From the perspective of ecosystem stability, the species composition in the NETP is relatively simple and has little ability to buffer external disturbance. Further detailed information on the topography, climate, and hydrology can be found in previous dendrochronological works (Shao et al., 2009; Yang et al., 2014).

**Table 1:** Descriptive statistics for the Qilian juniper chronologies developed in this study. The sample sites of DLH1–6, WL1–4, and TJ1 were obtained from Shao et al. (2009) and MNT, XDC, XHC, SBP, ZHG, SGX, and NDC from Yang et al. (2014). R_chron-PDSI_ refers to the correlation coefficient between tree-ring chronologies and June scPDSI(the self-calibrated Palmer Drought Severity Index (1850-2012 scPDSI dataset, http://www.cgd.ucar.edu/cas/catalog/climind/pdsi.html); Dai, 2011) during the study period. PMR refers to the percent of missing rings during drought years compared to all tree-ring width data for the four selected drought years. MS and AR1 represent mean sensitivity and first-order autocorrelation, respectively. AC, age class.

Elevation	Age class (year)	No. of trees	Mean ring	R_chron-PDSI_	MS	AR1	PMR (%)	Site codes
**width (mm)**
High	AC1 (100–300)	41	0.46	0.64	0.35	0.37	2.44	DLH3, DLH4, MNT, XDC, XHC
High	AC2 (301–500)	56	0.35	0.61	0.35	0.27	1.32	DLH4, MNT, XDC, XHC
High	AC3 (501–700)	42	0.29	0.62	0.38	0.26	2.84	DLH3, DLH4, MNT, SBP, ZHG, XDC, XHC
High	AC4 (700–900)	35	0.27	0.5	0.38	0.29	6.25	DLH3, DLH4, MNT, SBP, ZHG, SGX
High	AC5 (901–1,100)	43	0.27	0.62	0.41	0.16	5.43	DLH3, DLH4, SBP, ZHG, SGX, XDC
Low	AC1 (100–300)	31	0.35	0.64	0.43	0.26	14.5	DLH1, DLH2, DLH6, TJ1, WL1, WL2, WL4, NDC
Low	AC2 (301–500)	68	0.36	0.6	0.4	0.3	6.84	DLH1, DLH2, DLH5, DLH6, NDC, TJ1, WL1, WL3, WL4
Low	AC3 (501–700)	53	0.29	0.66	0.42	0.29	10.33	DLH1, DLH2, DLH5, DLH6, TJ1, WL1, WL2, WL3, WL4, NDC
Low	AC4 (701–900)	53	0.26	0.62	0.52	0.24	15.84	DLH1, DLH2, DLH5, DLH6, TJ1, WL1, WL3, WL4
Low	AC5 (901–1,100)	48	0.23	0.7	0.53	0.28	16.8	DLH1, DLH2, DLH5, TJ1, WL1, WL2, WL3, WL4

Each tree-ring width series was detrended with a 10-year cubic smoothing spline in the R-environment (R Core Team, 2017) using the function “detrend” in the “dplR” package (Bunn, 2008) to eliminate non-climatic trends and preserve higher interannual variability (Cook and Peters, 1981). This step transforms the raw tree-ring width sequence into a standard and dimensionless ring width index. A mean standard chronology (STD) was established using the biweight robust mean of the detrended ring widths of each age class (Cook, 1985) with the function “chron” for the overlapping period 1950–2000. The chronologies were characterized by mean ring widths, Gleichläufigkeit (GLK), mean sensitivity (MS), and first-order autocorrelation (AR1) (Fritts, 1976). GLK represents the similarity in signals between chronologies (Schweingruber, 1988). MS measures the mean percentage change in year-to-year growth variations. AR1 represents the influence of the previous year’s growth on the current year. MS and AR1 were calculated using ring width series, while GLK was based on the STD chronology.

### Selection of Extreme Drought Years Corresponding to Tree Growth Decline

Drought triggered by anomalously low precipitation and/or high temperature might yield exceptionally narrow tree rings over a large area in moisture-limited environments (He et al., 2018; Wang et al., 2015). The following two steps were applied to identify extreme drought years and ensure that they were primarily responsible for the sharp decline of tree growth (rather than other growth disturbances such as fire or insect pests).

**Figure 3:**
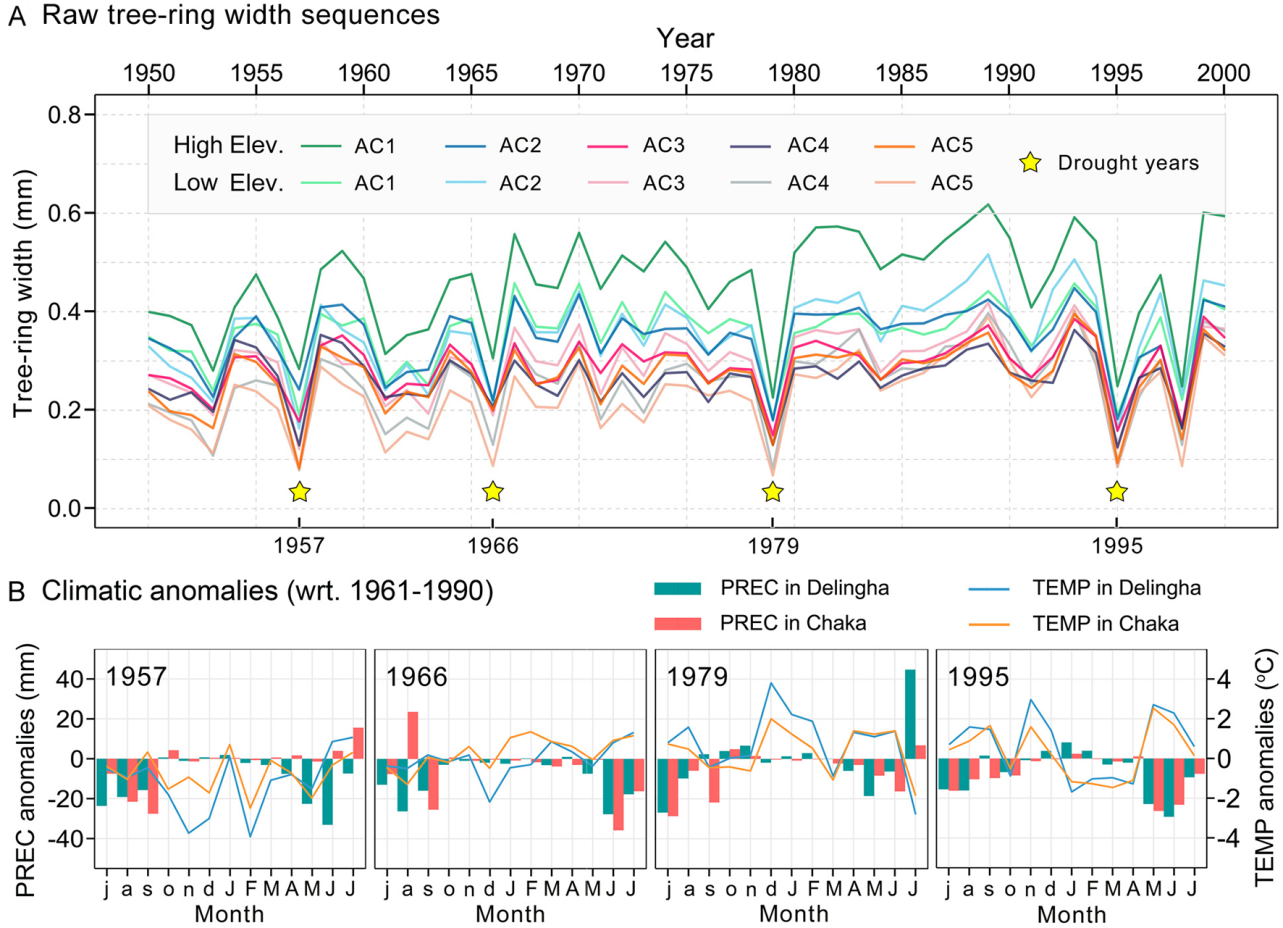
**(A)** Comparison of the mean tree-ring width series among five age classes and two altitudes. The yellow stars represent the four drought years selected in this study. **(B)** Climatic conditions during the four extreme drought years over the “annual water year” (here, July of the previous year to July of the current year). Months of the previous years are in lowercase and those of the current year in uppercase. “wrt”, with respect to.

1. Many previous dendrochronological studies in the adjacent area indicated that tree growth is primary restricted by the water shortage in June (Qin et al., 2013; Shao et al., 2005; Shao et al., 2010; Yang et al., 2014). Higher temperatures will enhance potential evapotranspiration, resulting in a further increase of moisture deficit during the same period (**Figure 3B**). Hence, we adopted the scPDSI sequence, which includes precipitation and surface air temperature (Dai, 2011), to identify drought episodes associated with tree growth decline. Firstly, we performed correlation analysis between the tree-ring chronology of each age class and scPDSI sequence, using the DendroClim2002 software (Biondi and Waikul, 2004), to assess drought responses of tree growth. Next, considering that the fastest radial growth period and main contribution to the annual ring width occurred in June (Gou et al., 2013; Zhang et al., 2018), we examined the scPDSI of each June during the study period and considered those years with values below 1.5 standard deviations from the mean as alternative drought years for subsequent analysis.
2. The “pointer year” (Schweingruber et al., 1990), referring to a year with notable growth variance occurring at the stand level, was calculated from the ring width index chronology of each age class. In this study, we only focused on growth declines associated with drought events. The pointer years were calculated using the R package “pointRes” (van der Maaten-Theunissen et al., 2015), with a threshold defined as 60% of tree-ring series showing a growth decrease of at least 30% compared to the average growth during the 2 preceding years.

We employed the drought years obtained from the first step to check the growth characteristics of tree-ring width series during these drought episodes and used the years with significant tree growth decline identified by the second step to control the number of extreme drought years ultimately adopted. Moreover, we ranked the intensity of the four drought years selected above based on precipitation and temperature anomalies during June, and ranking the driest year as fourth, the second driest year as third, and so forth, for further analysis.

### Quantification of Tree Vulnerability to Drought

The vulnerability of individual trees to drought events was quantified through the resistance (Rt), recovery (Rc), and resilience (Rs) indices adopted from Lloret et al. (2011). Resistance describes the magnitude of the growth reduction during a drought year compared to the pre-drought years and measures the ability of trees to “buffer” drought impacts. Recovery measures the growth difference between the post-drought period and the drought year and quantifies the capability of the tree to recover from drought disturbance. Resilience measures the difference in tree growth before and after a drought year and quantifies the capacity to reach pre-disturbance performance levels.

These three indices were calculated using the following equations:

Resistance (Rt) = Dr / PreDr

Recovery (Rc) = PostDr / Dr

Resilience (Rs) = PostDr / PreDr

The “PreDr” and “PostDr” values were calculated with the average tree-ring width during the 2 years before and after a drought event. The “Dr” variable refers to the tree-ring width for the drought year. All calculations follow the algorithm described above and were conducted using the raw ring width sequence on each individual tree. We used the 2-year period as the reference time interval for two reasons: first, we found that the growth of trees generally returns to its previous level within 2 years after the drought event, and second, this time scale allows full consideration of the lag effects of drought while avoiding interference with other disturbance factors (such as the next drought event). Considering that the relationship among these resilience indices is relatively intuitive (resilience = resistance × recovery), we only used the resistance and resilience indices for further analyses.

SEA was also utilized to evaluate tree growth response to drought extremes and, more importantly, to verify the results that we obtained above. SEA was applied to each age class by generating composites of tree-ring width indices from lag –2 to lag +2 years relative to the average of the previous 2 years before each drought event, to obtain the range of growth decline. A bootstrap resampling procedure (using 1,000 random sets) was applied to estimate whether tree growth in these drought years was statistically significantly different from the random sets of other years (lag +1 year). All the analyses were performed using R version 3.4.3 (R Core Team, 2017).

### Data Analysis

To facilitate the analysis, we performed cyclic elimination on the calculated resistance and resilience values by using the mean plus/minus 3 standard deviations rule (Limpert et al., 2001) to remove the presence of a very few outliers (less than five data points for each index), which we found to have no significant effect on the results. The differences in each index between three age classes and between two elevation gradients were assessed by the non-parametric Kruskal–Wallis analysis of variance (ANOVA) test (Kruskal and Wallis, 1952; unlike other regression methods, this one does not require the original data to be normally distributed) and the Dunn–Bonferroni test (Dunn, 1961) for *post hoc* comparisons.

The importance of age and elevation to resistance and resilience was assessed through a generalized linear model. In order to more accurately assess possible controlling factors of resistance and resilience, the sorted drought levels, latitude, and longitude, as well as tree age and elevation, were all added to our model. Ultimately, we operated the model with the data groups AC1, AC3, and AC5, and verified the accuracy of the model with the actual values from groups AC2 and AC4.

## Results

### Radial Growth and Tree-Ring Chronologies

Parallel radial growth patterns were detected among all age classes of Qilian juniper over the period 1950–2000 (**Figure 3A**). Whether at higher or lower sites, nearly all younger trees showed wider ring widths compared to older trees throughout the study period (mean ring width ranges from 0.46 to 0.27 mm for high altitude and 0.36 to 0.23 mm for low altitude). Moreover, trees growing at higher elevations showed wider ring widths compared to those of the same age at lower elevations. In particular, trees growing at lower altitudes or/and belonging to older age classes exhibited a greater proportion of missing rings during drought years than that of other tree individuals (**Table 1**). The mean AR1 of tree-ring width series was 0.27, showing that, during the study period, the growth conditions of the previous year had no significant effect on the tree-ring width of the current year. The MS of all trees exceeded 0.4, indicating a higher interannual variability of radial growth. The high GLK (0.87) among all chronologies suggests that the growth of trees within our study was highly consistent and that the chronologies are suitable for climate-growth analysis.

### Extreme Drought Years

Based on the selection methods described above, the drought years associated with notably slow growth of Qilian junipers are 1957, 1966, 1979, and 1995. It should be noted that rapid growth decline has also occurred in 1998, but it was probably due to other factors than drought and thus not included in the subsequent analysis as a drought event. These four drought years were ranked according to the precipitation and temperature anomalies in June (with respect to 1961–1990). Of these, the most severe drought year was 1995, followed in descending order by 1966, 1979, and 1957 (**Figure 3B**). Moreover, the correlation analysis results of tree-ring chronologies and scPDSI showed that the correlation coefficient in June was the highest during the whole hydrological year (**Table 1**); therefore, using the scPDSI value of June to identify extreme drought years is a reasonable approach.

### Changes of Resistance and Resilience Over Time

**Figure 4:**
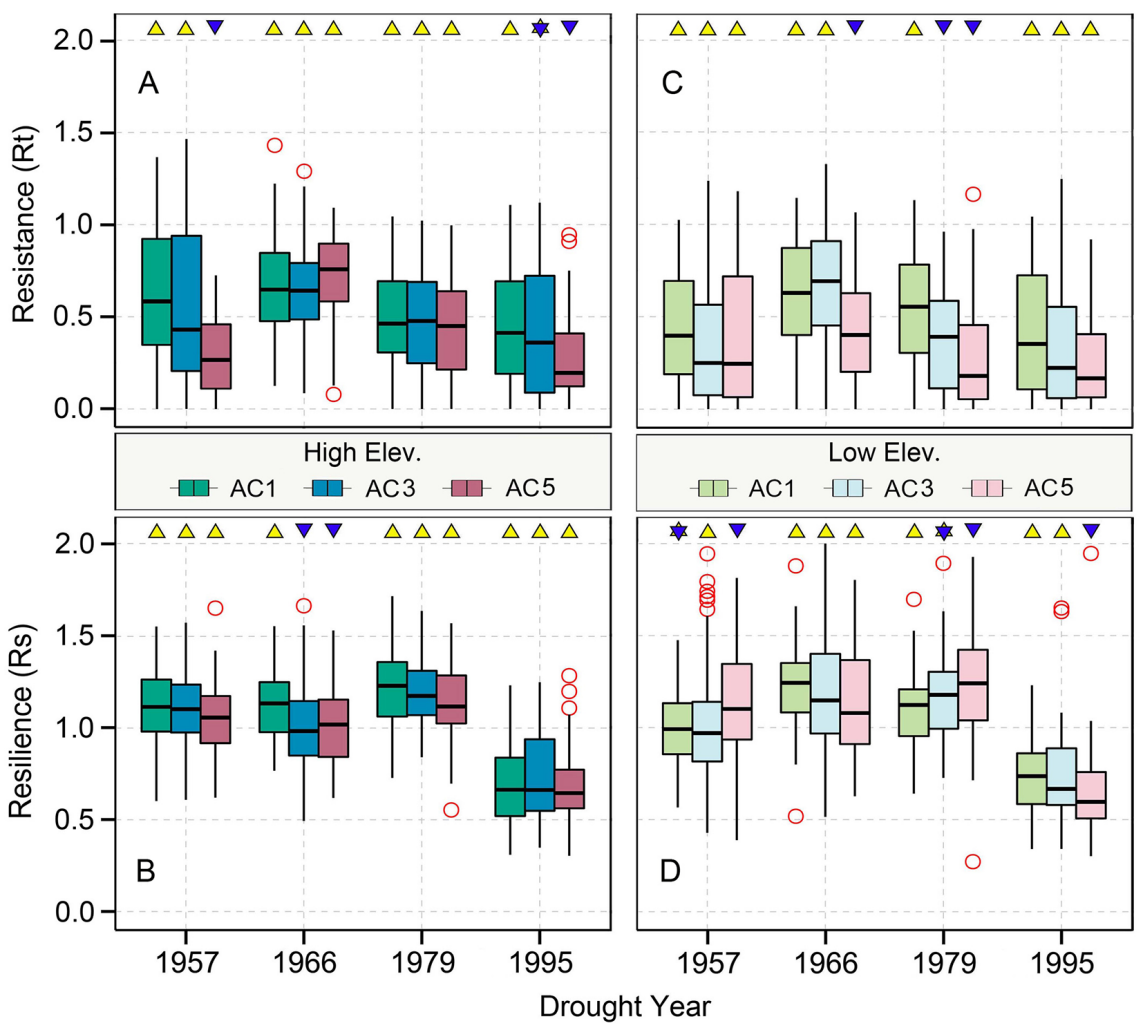
Age-related Qilian juniper responses to extreme drought years in terms of resistance **(A, C)** and resilience **(B, D)** of trees from higher and lower elevations, respectively. Yellow triangles and blue inverted triangles indicate differences among the three age classes for each index (Kruskal–Wallis analysis of variance (ANOVA) *post hoc* tests, *p* < 0.05). Red circles represent outliers, and vertical lines show the whiskers for the 5th and 95th percentiles of the data distribution.

**Figure 5:**
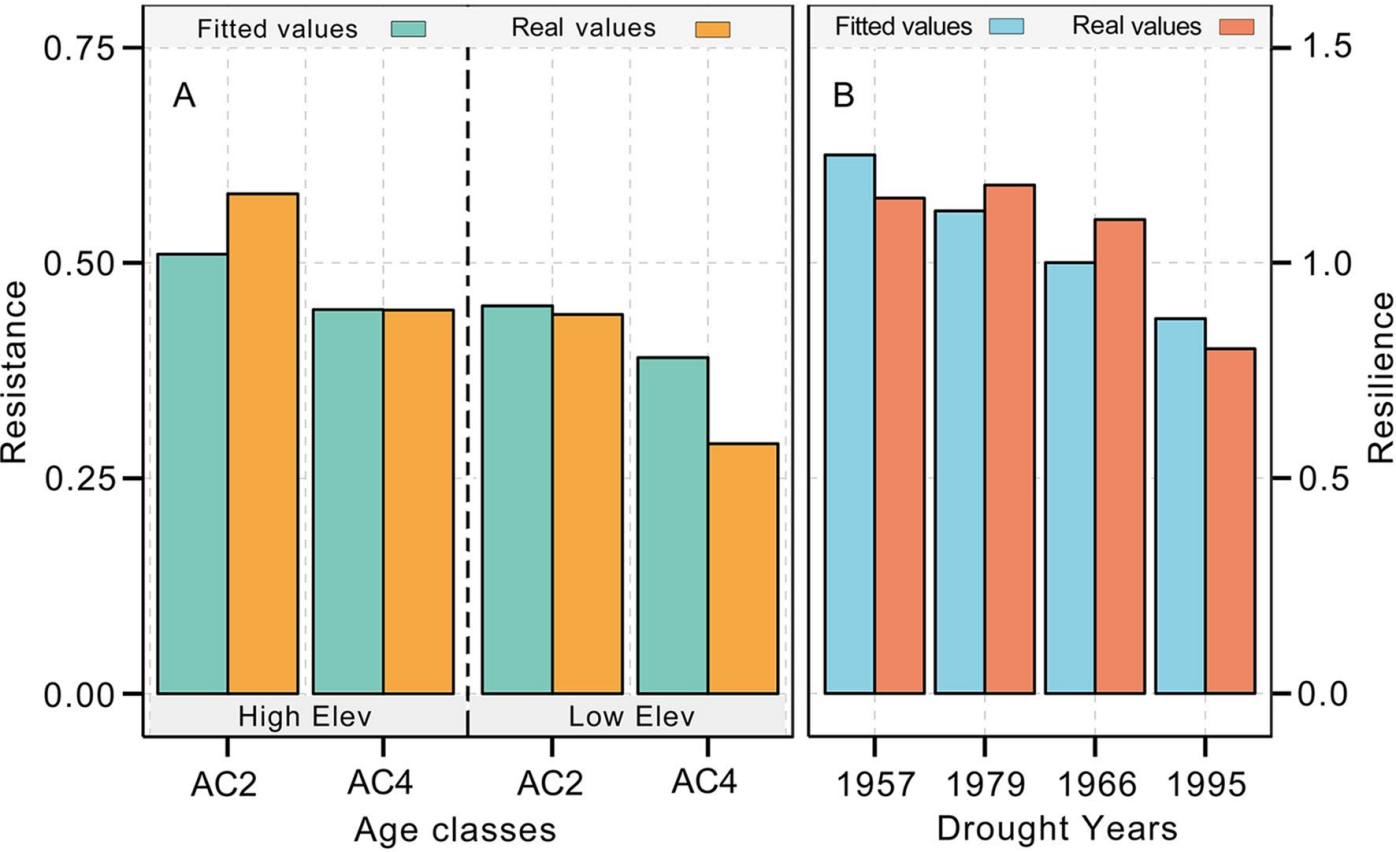
Comparison between the fitted resistance **(A)** and resilience values **(B)** and the real values. The regression equation is the generalized linear model generated by the age class 1 (AC1), AC3, and AC5 age groups of Qilian junipers. Please refer to **Table 1** and the main body of the text for the specific age classes of Qilian juniper.

The resistance of Qilian juniper to extreme drought for all age classes showed a gradually declining tendency, with the resistance in 1995 being the lowest among the four extreme drought years (–13%, –44%, and –6% for low-latitude trees and –21%, –43%, and –9% for high-altitude trees compared to 1957, 1966, 1979, respectively, based on the algorithm (Rt_1957_ − Rt_1995_)/Rt_1957_; **Figures 4A, C**). Regardless of the age and elevation, the resilience of almost all trees remained nearly constant during the three drought years of 1957, 1966, and 1979. However, a sudden drop in 1995 is evident (Rs decreased by 38% compared to the average of the previous three drought years; **Figures 4B, D** and **5B**), with 90% of all sample trees showing resilience values less than 1 and 65% less than 0.75. Thus, both resistance and resilience values in 1995 were the lowest among the four drought years.

### Comparison of the Radial Growth Responses in Different Age Classes

**Figure 6:**
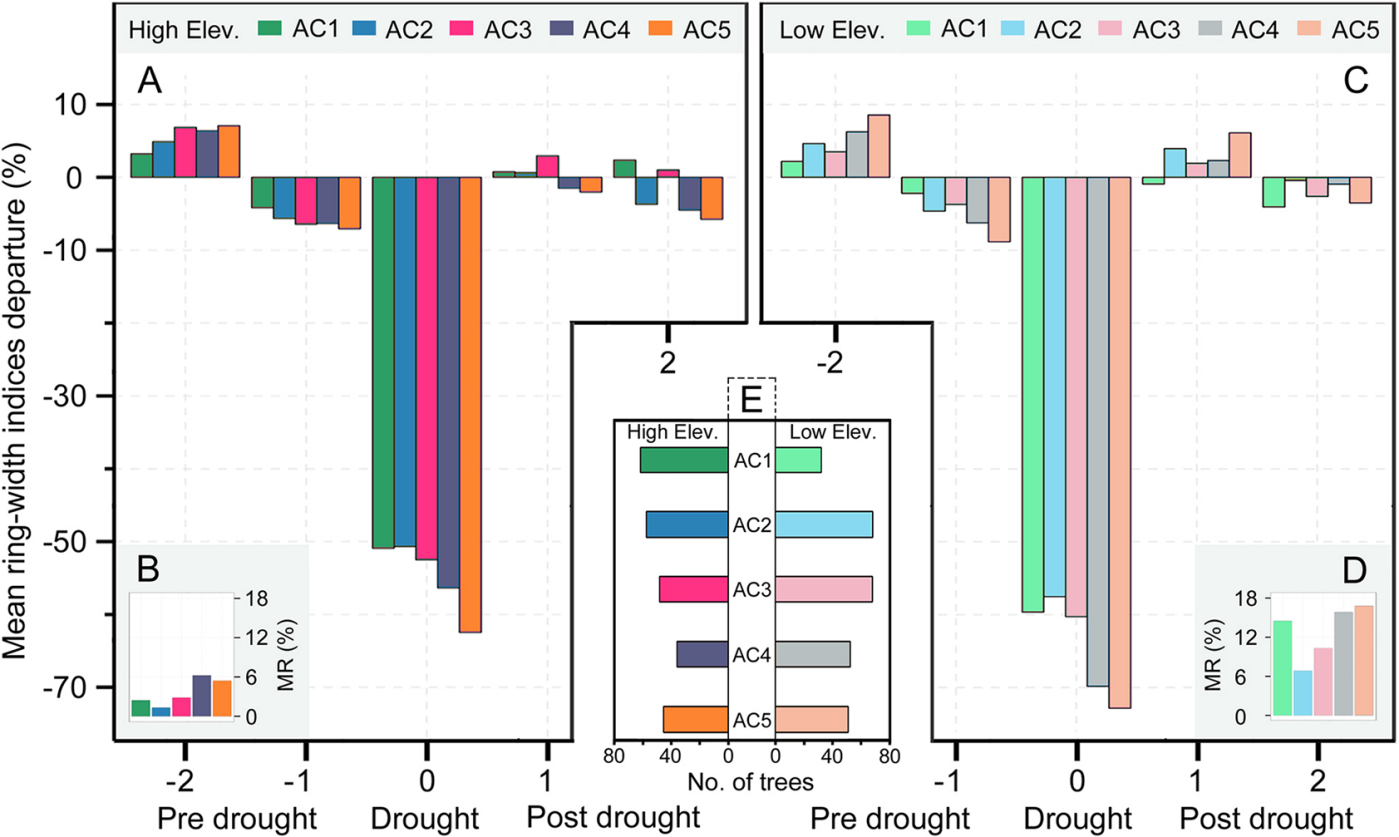
The percent of mean tree-ring width indices’ anomalies relative to the average values during the 2 years (lag-2 to lag-1 years) prior to each drought event for Qilian juniper at high **(A)** and low altitude **(C)**, respectively. The graphs in the lower corners **(B, D)** represent the percent of missing rings during drought years compared to all tree-ring width data for the four drought years. The middle lower panel **(E)** represents the age structure of the trees used in this study at high and low altitudes, respectively.

The resistance of Qilian juniper to extreme drought events showed a descending trend with increasing tree age (**Figures 4A, C**; **Table 2**). The oldest trees showed the lowest resistance during nearly all drought years (the average resistance of trees in AC5 was 11%/54% lower than in AC1 for high/low altitude; these differences were statistically significant in 1957 and 1995 for high altitude and in 1966 and 1979 for low altitude, with *p* < 0.05). Consistent results were obtained with the generalized linear model (**Figure 5A**). Likewise, the results from the SEA indicated that superposed tree-ring width indices during drought years were strongly reduced compared to the average of the previous 2 years and that the magnitude of the decrease was proportional to the age of the trees (**Figures 6A, C**). Furthermore, the proportion of missing rings during drought years in older Qilian junipers was higher than that of younger trees (**Figures 6B, D**), indicating that the older trees react more strongly to drought events. The resilience of Qilian juniper to drought events was somewhat less clear and did not change with increasing tree age (**Table 2**).

**Table 2:** The impacts of age, elevation, drought intensity, and latitude for the resistance and resilience of Qilian juniper to extreme drought events, calculated using the generalized linear models (GLMs), where DI = drought intensity, b = regression coefficient, SE = standard error, T = corresponding T statistic (for the partial test of H0: b(i) = 0), and p(T) = corresponding significance value.

Resilience indices	b	SE	T	p (T)
*Resistance*				
Intercept	0.55	0.53	1.04	0.30
Age	−0.0002	0.000027	−7.36	<0.001
Elev	0.00031	0.000057	5.44	<0.001
DI	−0.014	0.006	−2.28	0.02
Lat	−0.03	0.013	−2.33	0.02
*Resilience*				
Intercept	3.73	1.32	2.84	0.005
DI	−0.13	0.0066	−18.97	<0.001

### Effect of Altitude on Resistance and Resilience Variability

**Figure 7:**
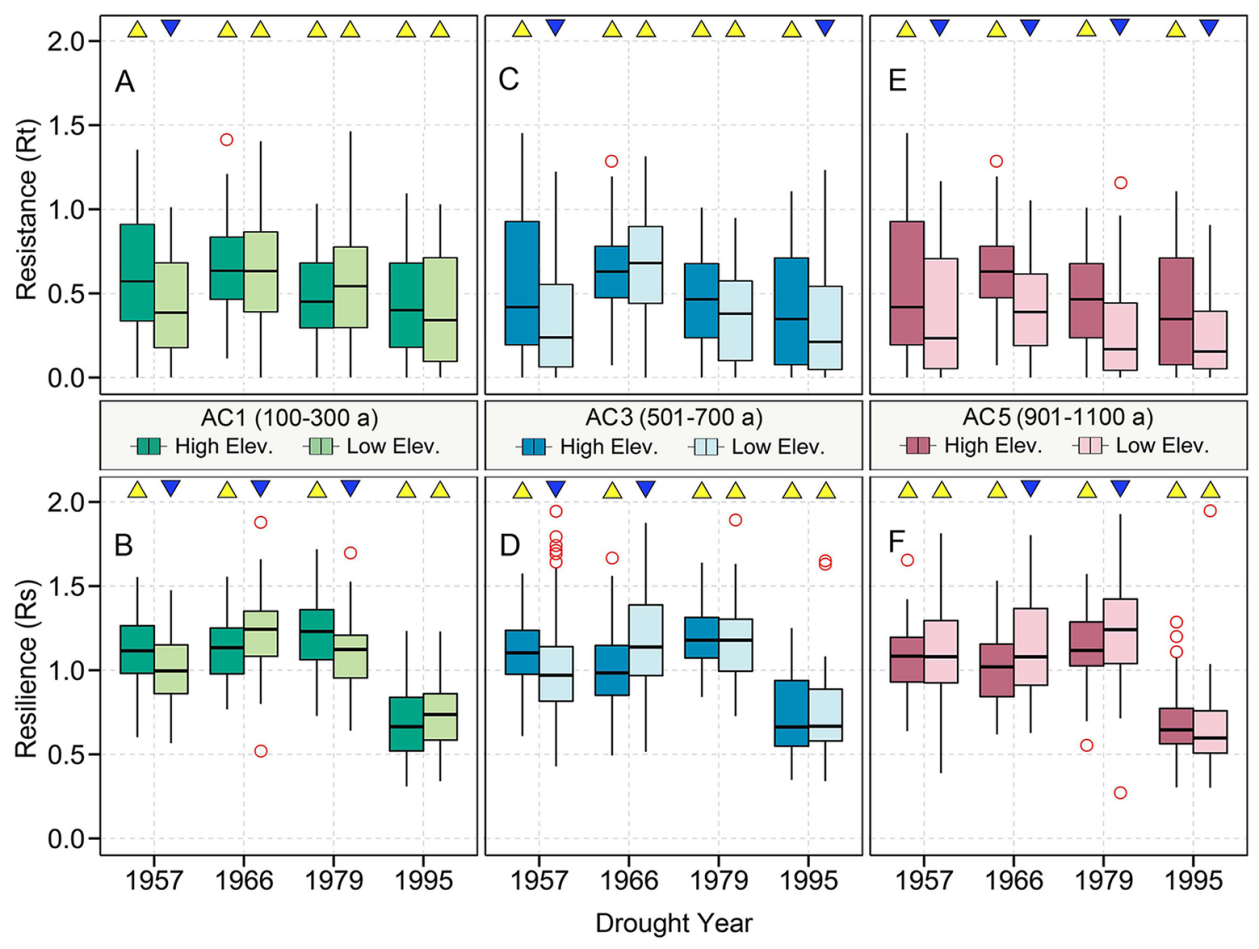
The effect of altitude on tree resistance **(A, C, E)** and resilience **(B, D, F)** to drought events among different age classes of Qilian juniper. Yellow triangles and blue inverted triangles indicate differences between high and low elevations for each index (Kruskal–Wallis ANOVA *post hoc* tests, *p* < 0.05).

Qilian junipers from lower-altitude sites showed smaller resistance values than those from higher-altitude sites within the same age class (**Figures 7A, C, E**; **Table 2**), which is especially evident in AC5 (**Figure 7E**). We note that the age structures of the sampled trees were similar at high and low altitudes (**Figure 6E**) and that comparison of resistance at different altitudes was performed within the same age group: hence, the difference in resistance between trees at different altitudes was not directly caused by the age effect. Furthermore, results of the SEA and the number of detected missing rings indicated that the magnitude of growth decline during drought events was greater at lower elevation (**Figures 6B, D**). Changes in resilience with altitude did not follow a distinct pattern (**Figures 7B, D, F**) for Qilian juniper, but trees with resilience > 1 accounted for 69% at higher altitude and 64% at lower altitude. The location (latitude and longitude) cannot explain the variations of resilience; while latitude did have an effect on resistance, its effect was much weaker than tree age and altitude.

## Discussion

### Age Effect on Drought Responses

Our results revealed varying resistance levels among the different age groups of Qilian junipers subjected to extreme drought events. These changes may be primarily associated with physiological characteristics as well as diverse drought influences on water/nutrient supply and demand relationships at different age stages. From the perspective of demand, older trees possess a more complex structure and much greater volume of aboveground woody tissues, with a thicker trunk and more abundant foliage, which increases the autotrophic respiration and consumes more carbon to meet the demand of normal life activities (Granda et al., 2017; Lucas-Borja and Vacchiano, 2018; Ryan and Yoder, 1997).

Regarding the supply, the lower hydraulic conductivity within the trunks and branches, coupled with the low level of photosynthetic capacity and stomatal conductance in older trees, may mainly explain their lower resistance to extreme drought events. In particular, the hydraulic resistance increases with tree age/height (Dawson et al., 1990; Ryan et al., 2006). For older Qilian junipers, their greater size means xylem fluid needs to be transported upward for a longer distance to reach the top/lateral branches and leaves, which requires higher tension in the xylem water column (Ryan and Yoder, 1997) compared to younger trees. When severe drought occurs during the growing season, this hydraulic architecture increases the risk of cavitation or embolism in the xylem of older trees (Hacke et al., 2001; Johnson et al., 2012; Savi et al., 2015; Tyree and Sperry, 1989), further impeding xylem hydraulic function and exacerbating the water stress. In response, blade stomata of these trees close earlier to prevent further loss of xylem water. This, in turn, prevents carbon dioxide uptake and causes a decrease in the photosynthesis capacity, thus limiting the carbon assimilation in older trees. These factors reduce the competitiveness of older trees under drought conditions and ultimately trigger carbon starvation and growth decline or even tree mortality (Dulamsuren et al., 2019; McDowell et al., 2008; Sevanto et al., 2014). Many studies have suggested that photosynthesis and stomatal conductance of old trees are lower than those of younger individuals (Grulke and Miller, 1994; Kull and Koppel, 1987; Schoettle, 1994), consistent with the above arguments. Therefore, older trees in moisture-limited environments with less water and nutrient supply are already closer to a critical point during normal years, and thus less resistant to extreme drought events.

Contrary to our results, a few studies have shown that older trees, with their well-developed root systems, can more easily absorb inorganic salts and water from a deeper and broader extent of the soil; this helps to buffer water stress during drought (Fritts, 1976; Krämer, 1996; Rozas et al., 2009; Wu et al., 2013). However, this effect was not observed in Qilian juniper at our study sites. Although root depth and lateral root extension increased with aboveground canopy size, root architectural traits are more closely related to water infiltration depth to allow more effective absorption of soil water in arid environments (Schenk and Jackson, 2002). Evidence supporting this view is the characteristic of Qilian juniper as a shallow-rooted species, with rooting depths ranging from 40 to 100 cm below the ground surface (Gou et al., 2013; Xu et al., 2011). Thus, while older Qilian junipers possess well-developed rooting systems compared to younger trees, their greater extent is mainly lateral and is not significantly deeper. Those well-developed roots of older Qilian junipers are produced to absorb water more effectively under normal hydrological conditions. However, based on the theory that smaller roots are more vulnerable to cavitation than stems (Sperry and Ikeda, 1997), it is these trees that are more exposed to the danger of cavitation during the dry periods, intensifying the poor water availability of older trees.

### The Influence of Altitude on Radial Growth Reactions

The change in altitude theoretically affects the distribution of heat and moisture resources. In general, thermal stress increases, whereas water stress decreases with altitudes in alpine environments (Cavieres et al., 2006; Gao et al., 2017; Wilson et al., 2005). Greater water availability at higher altitudes than at lower altitudes allows trees at higher altitudes not only to grow more rapidly under normal hydrological years but also to more effectively buffer the adverse effects of water deficits during drought years. Our results showed that the average tree-ring width at lower altitudes was invariably narrower, and that resistance to drought was significantly lower, than those at higher altitudes, consistent with the results discussed above.

The percentage of missing rings, which can reveal the severity of drought stress on tree growth and which is inevitably linked to tree mortality (Liang et al., 2016a), was higher at lower elevations in our study. The radial growth of all tree samples used in this study was mainly limited by water deficit. Given that trees at different elevations will suffer different degrees of drought, due to changes in the distribution of precipitation and temperature along the altitudinal gradient during the same drought event, this may indicate that environmental conditions at lower altitudes are less favorable (lower water availability) than those at higher altitudes, prompting the trees in these areas to react more strongly to the extreme drought events. It is worth noting that the relatively higher temperatures at lower elevations could theoretically deepen the seasonal thaw depth of the permafrost in summer, thus alleviating the water deficit in these areas to some extent. However, the maximum thaw depth of the permafrost in this region is beyond 1.5 m (Huang et al., 2011), while the soil layer and the roots of Qilian juniper are mainly concentrated within the uppermost 1 m (Gou et al., 2013; Xu et al., 2011). Therefore, the changing contribution of summer permafrost melting to soil moisture along the elevation gradient does not ultimately change the pattern of lower-elevation sites facing greater moisture deficits.

The slightly increasing precipitation during the study period might have promoted tree growth at higher elevations, leading to increasing canopy cover and stem density; this could have intensified the competition among trees for soil moisture, causing a higher frequency of missing rings as observed by Liang et al. (2016a) in the central Qilian Mountains. However, this effect was not seen in our study, presumably because the canopy density of Qilian juniper within our study sites (**Figure 2**) is far less than that in the Qilian Mountains (0.3 *vs.* 0.6–0.8) (Qin et al., 2013; Liang et al., 2016a), and there is no distinct intraspecific competition among trees at higher altitudes.

### Climate Threshold–Related Growth Decline

Our results demonstrated that the resilience of Qilian juniper to extreme drought is not directly related to the elevation, age, latitude, or longitude of trees, but instead showed a significant negative correlation with the intensity of drought events. The mean resilience value fluctuated around 1, as shown in 1957, 1966, and 1979, indicating that these droughts did not seriously injure the Qilian juniper; indeed, the trees showed rapid recovery to pre-drought growth levels after the droughts. Resilience values significantly lower than 1 (e.g., in 1995) suggested that drought intensity was particularly high, with a strongly adverse impact on these trees, such that the majority of trees had not yet returned to their pre-drought growth status within 1 to 2 years following the drought episodes. Some of the above variability may also be attributed to patterns of water deficit at regional scales.

The average anomalies of precipitation and temperature (**Figure 3B**) showed that a strong negative precipitation anomaly was superposed on a positive temperature anomaly during June 1995 at both Delingha and Chaka meteorological stations (–29.3 and –23.3 mm for June precipitation anomaly and +2.3°C and +1.7°C for June temperature anomaly in Delingha and Chaka, respectively), far exceeding the magnitude of temperature and precipitation anomalies in 1957, 1966, and 1979. Therefore, the 1995 growing season was the driest for Qilian juniper during the study period. Indeed, 1995 was documented as one of the driest years on the NETP in other studies (Fang et al., 2012; Tian et al., 2007; Liang et al., 2016a). Thus, the Qilian juniper samples suffered from more severe water shortage in 1995 than during any of the other three drought years. The 1995 drought was associated with significant growth decline and longer recovery period. Our results indicate that almost all trees used in our study survived all four extreme drought events. However, in making this interpretation, we may have overlooked the important aspect that all tree samples we used were from living trees; consequently, those trees that had died after a specific drought event were not included in subsequent samples. There are precedents for tree mortality due to high-intensity droughts in many parts of the world (Allen et al., 2010; Anderegg et al., 2013; McDowell et al., 2008). Therefore, we can infer from our results that if a very severe drought event occurs during the growing season (like that in June 1995), and if it exceeds a level for some trees, then those trees will die first, while the other more drought-tolerant individuals may survive after the drought.

Unfortunately, such thresholds are rarely studied through dendrochronology, and it is difficult to obtain robust estimates. Theoretically, it is possible for resilience to equal zero; this can be interpreted as the failure of some trees to form a radial increment during the 2 or more years after the extreme drought (i.e., missing rings) and includes the possibility that the tree died. A resilience of zero was not observed in our study, which may be due to the inherent weakness of the evaluation method that we adopted (Lloret et al., 2011). Therefore, a priority for future work will be to seek these dead/dying tree samples that are most vulnerable to drought events and compare their data with surviving trees to analyze their growth status before and after drought, with the aim of better understanding tree resilience to drought and the threshold of tree mortality.

### Considerations of Future Forest Variability Under Global Warming

The increasing temperature and precipitation associated with global warming in our study region during recent decades, coupled with a possible carbon dioxide fertilization effect (Piao et al., 2012), have been beneficial to the radial growth of Qilian juniper on the NETP. This has been demonstrated by the slightly upward trend in tree-ring width sequences during the study period. However, the slight rise in precipitation has not changed the overall status of water shortage on the NETP. Furthermore, a growing number of studies have shown that greater variability of precipitation under global warming will enhance the intensity and frequency of extreme drought events (Kim and Byun, 2009; Trenberth et al., 2014). This will undoubtedly be a greater test for Qilian junipers growing in the arid Tibetan Plateau of inner Asia, since our results showed that the recovery ability of Qilian juniper after drought events is closely related to the drought intensity.

In addition, there is general consensus that the growth of Qilian juniper over broad areas of the lower/middle altitudes is mainly limited by water availability. However, Zhang et al. (2015) directly addressed this issue by collecting Qilian juniper samples in the uppermost 20% of the forest belt and concluded that low temperature rather than drought limits the radial growth of Qilian juniper at the upper tree-line (∼4,250 m a.s.l., higher than in all of the other related studies). With continued global warming, trees growing near the upper tree-line may benefit from increasing temperatures as thermal conditions become less restrictive on tree growth, thereby leading to an upward migration of the upper tree-line (Liang et al., 2016b). According to our results, the younger Qilian junipers at lower altitudes are the most vulnerable groups to drought events, while the younger individuals at higher altitude are more resistant. Ongoing global warming and associated expansion of the forest belt to higher elevations will have two main consequences: one is that the warming process is equivalent to expanding the range of low-elevation Qilian junipers, causing the older trees in these areas to be exposed to more severe water stress conditions; the other is to increase the rate of forest recruitment at high altitudes, where younger trees are more resistant to drought and are under less severe drought stress. However, if this trend continues, the upper tree-line will eventually be constrained by other factors (e.g., terrain, or the height at which precipitation no longer increases with elevation). From that time onward, the forest belt will no longer be able to expand upward, yet the intensity of water shortage would continue increasing under ongoing global warming, which is equivalent to placing a larger number of trees into the “lower-altitude environment” (warmer and drier), which would have an adverse effect on local forest ecological security.

## Conclusions

We studied Qilian junipers that are widely distributed across the NETP to explore the vulnerability of different tree individuals to severe drought events. In conclusion, we found that drought extremes have a significant adverse impact on the radial growth of Qilian juniper. The effects of age and elevation both strongly affect the ability of Qilian junipers to cope with drought extremes: specifically, older trees from lower elevations are more vulnerable to drought and may even be exposed to a higher risk of mortality should water shortages become aggravated in the future. Meanwhile, the resilience of Qilian junipers to extreme drought showed no connection with tree age, elevation, latitude, or longitude, but was closely correlated with drought intensity. Thus, we can reasonably speculate that, although all the Qilian juniper samples used in this study had successfully recovered from the four selected drought events, there are some very vulnerable tree individuals (e.g., some of the oldest trees at lower altitudes) that may already be dead if the intensity of one of those drought episodes exceeded their endurance limit; these individuals will not have been included in our samples. This is important in the context of global warming, since increased precipitation variability may enhance the intensity and frequency of extreme drought events. Our study revealed which tree individuals are more vulnerable to drought extremes, enabling identification of those trees most seriously affected by drought so that their growth and even death processes can be monitored. This would not only provide the possibility of estimating threshold conditions for tree mortality but also enable us to better understand the dynamics of forests and even ecosystems across the NETP under the background of climate warming.

## Data Availability Statement

The datasets generated for this study are available on request to the corresponding author.

## Author Contributions

BY and XW designed the study. XW analyzed the data and wrote the first version of manuscript. XW, BY, and FL revised the manuscript and approved the submitted version.

## Funding

This study is supported by the National Nature Science Foundation of China (NSFC grant nos. 41520104005 and 41325008), and by the Belmont Forum and JPI-Climate Collaborative Research Action “INTEGRATE” (NSFC grant no. 41661144008). FL is supported by the Swedish Research Council (Vetenskapsrådet, grant no. 2018-01272).

## Conflict of Interest

The authors declare that the research was conducted in the absence of any commercial or financial relationships that could be construed as a potential conflict of interest.
